# Comparison of High-Speed Polarization Imaging Methods for Biological Tissues

**DOI:** 10.3390/s22208000

**Published:** 2022-10-20

**Authors:** Xianyu Wu, Mark Pankow, Taka Onuma, Hsiao-Ying Shadow Huang, Kara Peters

**Affiliations:** 1 Department of Mechanical and Aerospace Engineering, North Carolina State University, Raleigh, NC 27695, USA; 2 School of Mechanical Engineering and Automation, Fuzhou University, 2 Xueyuan Rd., Fuzhou 350116, China; 3 Photron Limited, Kanda Jinbo-Cho 1-105, Chiyoda-Ku, Tokyo 101-0051, Japan

**Keywords:** high-speed imaging, polarization imaging, mechanical testing, tissue properties

## Abstract

We applied a polarization filter array and high-speed camera to the imaging of biological tissues during large, dynamic deformations at 7000 frames per second. The results are compared to previous measurements of similar specimens using a rotating polarizer imaging system. The polarization filter eliminates motion blur and temporal bias from the reconstructed collagen fiber alignment angle and retardation images. The polarization imaging configuration dose pose additional challenges due to the need for calibration of the polarization filter array for a given sample in the same lighting conditions as during the measurement.

## 1. Introduction

Recent advances in high-speed cameras have created new possibilities to apply polarization state imaging to dynamic loading cases [1,2,3,4,5]. Quantitative approaches for calculating the properties of local principal stresses within materials without fringe unwrapping have also evolved, which are commonly referred to as “dynamic photoelasticity.”For these approaches, a series of images are collected at different orientations of the optical polarization state generator. The polarization angle of orientation and relative retardation are calculated at each point in the field of view from these multiple polarization state images. Ramesh et al. [6] and Patterson [7] provided excellent reviews of dynamic photoelasticity. For high-speed dynamic events, the challenge is to collect the multiple phase images sufficiently fast.

In general, there are two different strategies to increasing the acquisition speed of the polarization state data at each pixel. The first is to use a classical optical configuration with a polarization state generator in front of the specimen and a polarization state analyzer between the specimen and the camera. The rotating element of the polarization state generator is then rotated sufficiently fast, generating two polarization and retardation maps per rotation, and the camera frame rate set sufficiently high to collect multiple images per rotation of 180${}^{\circ }$. Early demonstrations of the method used four to eight individual phase shifted images per measurement [8,9,10,11], although the minimum number required is three [12].

The second approach is to split the camera pixel array into different regions, divided into different polarization angles. Early versions of this method split the field of view into different beam paths with beamsplitters and projected them onto to distinct cameras [8]. Later advances created a polarizing filter array that is integrated with the camera imaging sensor, such that individual pixels measure different polarization components simultaneously. The most common approach is to create a 2 by 2 array of polarizers at 45${}^{\circ }$ relative orientations. Four individual pixels are then used per measurement point in the polarization angle and retardation images [2,3,4,5]. Onuma and Otani [2] developed an integrated, 2 by 2 pixelated polarizer array with this same optical arrangement and a multi-channel parallel read out to achieve a sampling rate of 1.3 MHz.

The rotating polarization state generator approach has a higher spatial resolution, as the full pixel resolution is preserved; however, the temporal resolution is lower than the camera’s frame rate, since multiple images are obtained per pixel to resolve the polarization and retardation images. When *N* polarization states are used, the temporal resolution is reduced by a factor of *N*. In addition, since the multiple polarization states are used to calculate a single alignment and retardation image, rapid changes in these properties in time can create a phase-lag in the images [1,13]. The polarizing-filter-array approach has a higher temporal resolution, as only one measurement in time is needed for each image; however, the number of effective imaging pixels is reduced by the same factor of *N*. The second approach is simpler to implement in a laboratory environment, since no rotating elements are required; however, it can be more complex in manufacturing because of the polarizing filter array.

Both approaches to dynamic polarization imaging have been successfully implemented by researchers for biological tissue samples. Photoelasticity, combined with phase-stepping methods, have been applied to these materials to determine the state of collagen fiber alignment (polarization microscopy). For these applications, the principal material angle and retardation correspond to the orientation and strength of the collagen fiber alignment. Quinn and Winkelstein [14] developed an imaging system with a rotating polarizer to achieve alignment images at 25 Hz. Wu et al. [1,13] later extended the acquisition speed to 10 kHz using a polarizer rotating at 5000 RPM and a sequential image analysis method. Gruev et al. [5] implemented a polarizer array of 1000 by 1000 pixels, using monolithically integrated aluminum nanowire optical filters, with four different orientations offset by 45${}^{\circ }$. The optical slow axis orientation and retardation of the samples were collected at up to 40 Hz [3,4,5].

At relatively high imaging speeds, the need for uniform lighting at intensity levels, sufficient for the short image acquisition times, becomes a significant challenge. Many biological tissues display a high level of birefringence during loading, for example, due to aligned collagen fibers, but also nontrivial amounts of inherent scattering. The inherent material scattering in tissues, combined with the short exposure time at high-speed imaging rates, can lead to low signal-to-noise ratios in the images. In addition, wide variations in material properties can exist over the region of interest, further degrading the imaging performance. Finally, these materials often deform considerably during dynamic loading, which makes tracking of material pixels between two consecutive images difficult.

In this study, we applied the polarization filter approach to dynamic polarization imaging of polymer (low-scattering) and tendon-to-bone insertion (high-scattering) specimens during dynamic, tensile loading at 7000 frames per second (fps). The results are compared to previous measurements (at similar frame rates) of similar specimens using a rotating polarizer imaging system. By comparing the results of the reconstruction of the collagen fiber alignment and retardation (strength of alignment), we evaluate the relative merits and issues between the polarization filter and rotating polarization state generator approaches for high-speed imaging of these materials under high deformation.

## 2. Experimental Methods

### 2.1. Imaging Method

**Figure 1:**
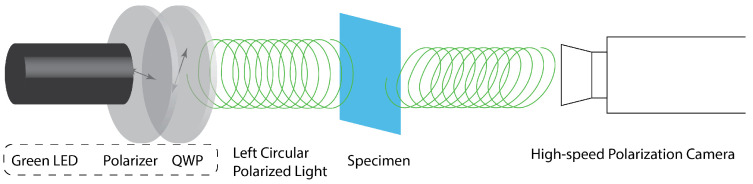
Schematic of the high-speed polarization camera’s optical train.

A schematic of the optical train used to measure the specimen birefringence parameters is shown in Figure 1. The setup uses a circular polarized light beam to illuminate the specimen and a high-speed polarization camera to record the light intensity of the output light beam. At the input side of the optical train, high-power LED sources were used to illuminate the specimen. For tests performed on the polycarbonate specimen, a 532 nm wavelength LED light source installed with a linear polarizer and a QWP was used to create a left circular polarized light beam to illuminate the specimen. For the tests performed on tendon-to-bone insertion specimens, this LED source was not sufficient to transmit enough light through the biological specimens. Therefore, the light source was replaced with a 520 nm LED optical fiber illuminator (SLG-55-G-0-1, Revox, Sagamihara, Japan), which produced a sufficient intensity to pass through the tendon-to-bone insertion region of a sliced specimen. The maximum luminous flux of the LED optical fiber illuminator is 2100 lm; the power of the LED is approximately 37 W. Waveplates were installed in front of the high power LED to create a left-handed, circular polarized light beam for measurement.

**Figure 2:**
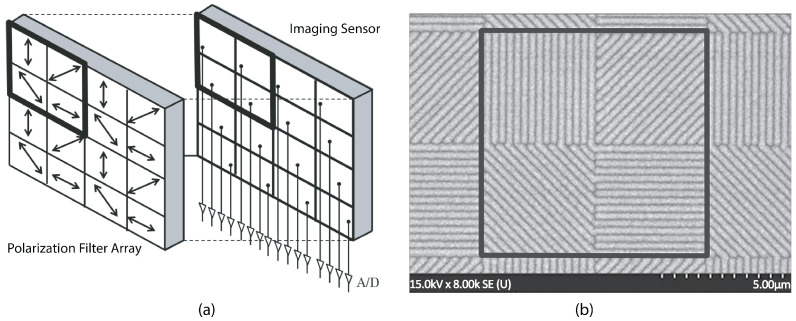
(**a**) Schematic of the basic structural unit of the polarization sensor array. (**b**) SEM image of four neighboring pixels with a micropolarizer array.

In the high-speed polarization camera (CRYSTA PI-1P, Photron Inc., Tokyo, Japan, 12 bit), the image sensor is integrated with an array of 2 × 2 photonic crystal polarization filters at four different orientation states, 0${}^{\circ }$, 45${}^{\circ }$, 90${}^{\circ }$ and 135${}^{\circ }$, as shown in Figure 2. The image sensor has a parallel readout of a multi-channel analog to digital converter (A/D) specialized for 2D polarization detection [2]. Since multiple readout circuits are connected to multiple pixels, the electrical charges are read out in parallel, permitting a high sampling rate up to 7 kHz for the full frame and 1.55 MHz for a smaller region of the image sensor. The pixilated polarizer array was made from a photonic crystal, fabricated with electron beam lithography, bonded directly to the image sensor, to decrease vibration-induced noise. More details on the performance validation of the polarization imaging system can be found in [2].

The light intensities in each 2 by 2 array of polarization filters were used to calculate the alignment angle, $\alpha_{s}$, and retardation of the specimen, $\phi_{s}$, at each time step, using

(1) $$\alpha_{s}=\frac{1}{2}\tan^{-1}\left(\frac{I_{90^{\circ }}-I_{0^{\circ }}}{I_{45^{\circ }}-I_{135^{\circ }}}\right),$$

(2) $$b_{s}=\lambda \sin^{-1}\left(\frac{2\sqrt{{\left(I_{90^{\circ }}-I_{0^{\circ }}\right)}^{2}+{\left(I_{45^{\circ }}-I_{135^{\circ }}\right)}^{2}}}{I_{0^{\circ }}+I_{45^{\circ }}+I_{90^{\circ }}+I_{135^{\circ }}}\right),$$

where $I_{0^{\circ }}$, $I_{45^{\circ }}$, $I_{90^{\circ }}$ and $I_{135^{\circ }}$ are the light intensities measured at the four pixels covered by the polarization optical filters [2]. The measurement dynamic range of $\alpha_{s}$ was $\pm 90^{\circ }$, and that of $b_{s}$ was 0 to λ/2, where λ is the illumination light’s wavelength.

The high-speed polarization camera provides a resolution of 1024 by 1024 pixels; hence, the resolution of the generated alignment and retardation images is 512 by 512 pixels. For the tests on the polycarbonate specimen, the high speed camera was operated at 3000 fps. However, for tests on the tendon-to-bone insertion specimens, the material displacement at each time step was greater, so the high-speed camera was operated at 7000 fps.

Prior to testing, it was necessary to calibrate the imaging sensor array for the specific optical train. This was performed prior to inserting the specimen. The calibration determines the range of expected intensities for each pixel and is performed by using retarders with known retardation values.

### 2.2. Specimens

The high-speed polarization camera measurement system was first applied to specimens with low levels of inherent material scatter (polycarbonate) and then to highly scattering biological specimens (tendon-to-bone insertion), both under rapid tensile loading. These two specimens types were chosen because their visualization with high-speed polarization imaging had been previously demonstrated by the authors using a rotating polarizer system [1,13].

**Figure 3:**
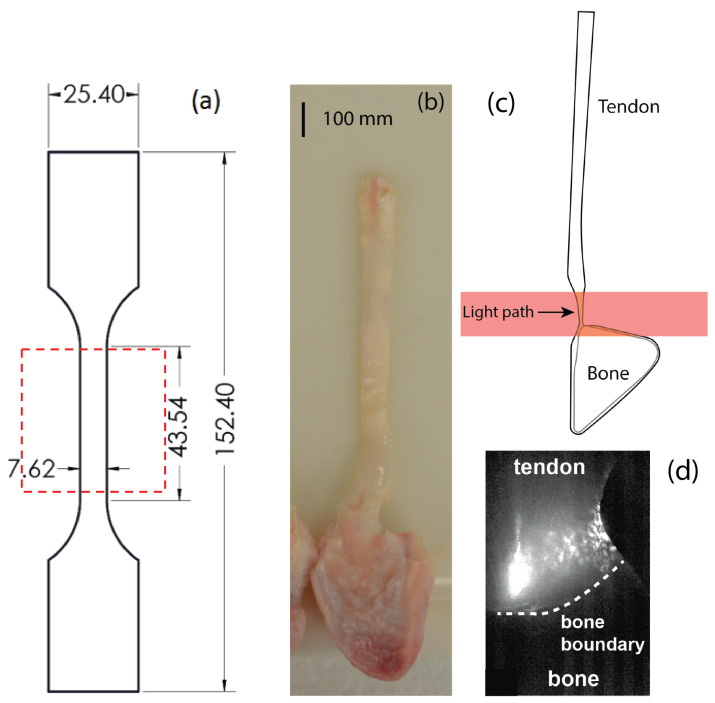
(**a**) Dimensions of the polycarbonate specimens. Dimensions are in mm. The thickness of each specimen in tendon-to-bone insertion region was 0.508 mm. Region of interest for the imaging is shown in the red box. (**b**) Photograph of a tendon-to-bone sample prior to thinning. (**c**) Schematic showing the thinned insertion region after slicing and the light propagation direction through the sample. (**d**) Camera intensity image of tendon-to-bone insertion region of interest.

The uniform-thickness polycarbonate dogbone specimens were cut using a laser cutter. The dimensions of the specimens are shown in Figure 3a. A small notch was created in the middle of the sample on one side to induce a crack and specimen failure during dynamic testing. As load was applied to these specimens, the polarization state alignment angle followed the orientation of the slow principal axis due to the applied stress.

Next, biological specimens with much larger amounts of inherent material scattering were tested. Tendon-to-bone insertion specimens, shown in Figure 3b, were tested using the same polarization camera arrangement. In biological-tissue-based specimens, the collagen fibers act as a rotating polarization element. The alignment axis therefore follows the orientation of the collagen fibers, which realign during dynamic loading, and can yield significant information on the behavior of the material [15]. The insertion region is the transition region between the flexible tendon and the rigid mineralized bone region. While the properties and orientation of the collagen fibers varied throughout the insertion region, they were uniform through the thickness of the specimen, making these ideal specimens for transmission polarization imaging [16].

Digital flexor tendon-bone units were removed from porcine forelimbs procured from a local abattoir. The raw tendon-to-bone samples were first frozen to −20${}^{\circ }$ using a cryostat. Then, a microtome was used to slice approximately 20 μm thick tissue layers from each surface. Reducing the specimen’s thickness improves the light intensity arriving at the high-speed camera. The specimen thickness was optimized based on previous drop-tower load testing of tendon-to-bone insertion specimens by the authors [13].

### 2.3. Impact Loading

**Figure 4:**
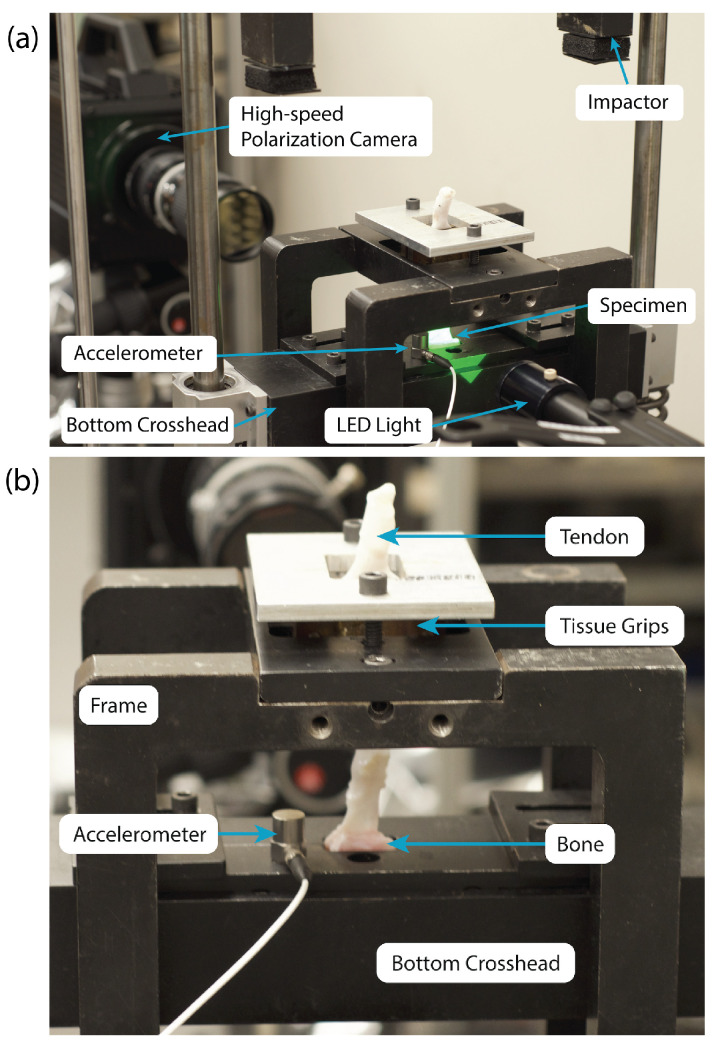
(**a**) Photograph of the high-speed polarization camera setup and modified drop tower. (**b**) Closeup of a tendon-to-bone insertion specimen in grips.

Both specimen types were mounted in a drop tower to apply dynamic tensile loads to the specimen. Figure 4 shows a tendon-to-bone insertion specimen mounted in the drop tower with the tendon exiting the top of the mounting frame (white tissue). To load the specimens, an impactor was released from above the specimen, which contacted the bottom crosshead and displaced it downwards, loading the specimen with tension. By adjusting the drop height, the maximum loading rate on the specimen was adjusted. Two springs were mounted under the bottom crosshead to remove any preloading from the sample to ensure each material was starting from a zero stress state.

For the polycarbonate specimens, standard flat grips were used to hold them. For the tendon-to-bone insertion specimens, specially designed curved, textured grips were used to clamp the tendon end of the specimen, which were attached to the frame of the drop tower. The tendon end was first threaded through a hole in the crosshead, so that the crosshead pushed down on the bone end of the specimen under tension to load it during the impact.

Since the position sensor’s data were not correlated with the high-speed polarization camera images during testing, the first frame in the camera video that showed specimen motion was set to be 0 ms as the time reference. Therefore, *t* = 0 is the start of an impact event. The displacement of the impactor, $x_{i}$, was measured throughout the fall of the impactor using a non-contact linear position sensor, from which the velocity of the impactor, $v_{i}$ was calculated. The loading displacement rate on the specimen is calculated at any time v(t) as

(3) $$v\left(t\right)=\frac{m_{i}}{m_{c}}\left[v_{i}\left(t\right)-v_{if}\right]-2\frac{K}{m_{c}}\int_{0}^{t}\left[x_{i}\left(t\right)-x_{i}\left(0\right)\right]dt$$

where $m_{i}$ = 10.84 kg and $m_{c}$ = 5.29 kg are the masses of the crosshead and impactor, respectively, $v_{if}$ is the freefall velocity of the impactor before the impact event and *K* = 20.458 kN/m is the stiffness of each of the two springs to decelerate the crosshead [13].

## 3. Results

### 3.1. Polycarbonate Specimens

**Figure 5:**
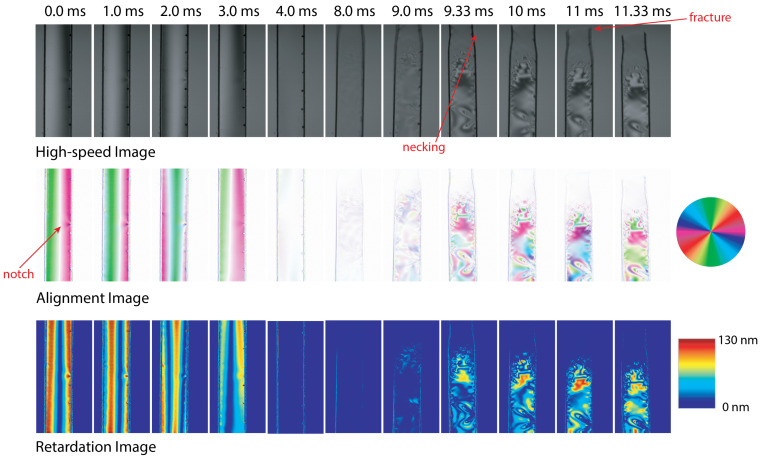
High-speed images recorded for a polycarbonate specimen using the high-speed polarization-filter-array camera and obtained optical axis alignment angle and retardation images.

Eight samples were tested to capture the responses of the polycarbonate samples. The results were comparable between samples. The data from one representative test are presented in this section. Selected video images, alignment angle maps and retardation maps for this specimen are shown in Figure 5. The video images were calculated based on the average of the light intensity measured by the four sensor pixels and converted to grayscale.

The loading began at 0 ms with increased tensile loading of the specimen until failure at approximately 11 ms. The specimen was loaded under tension along the vertical axis. Generally, there are three different periods that can be seen in the high-speed images. For 0 to 4 ms, the alignment angle images and retardation images show vertical line patterns from right to left. Prior to failure of the specimen, the alignment images were expected to show a uniform angle aligned with the horizontal direction (the slow axis of the tensile specimen). The vertical lines in the initial loading frames were due to the misalignment of the sample in the grips. The specimen was placed in the grips without pre-loading and was rotated relative to the light beam, in the order of a few tens of nanometers of displacement. Once loading was applied to the specimen, it underwent rigid body rotation to align with the loading axis. This rotation appeared as an apparent alignment angle retardation change until the stress induced birefringence was greater than this effect. As the load was increased, the specimen rotated until it was aligned, and the loading was pure tension, by 4.0 ms. A similar phenomenon was observed using the rotating polarizer method on similar specimens [1]. The location of the pre-cut notch can also be seen in these images.

After 4 ms, the specimen was under a uniform state of stress. However, the alignment images from 4 to 8 ms appear faded to a primarily white color. This fading was due to the fact that for the majority of sensor pixels, the calculation of alignment angle was unresolved, and therefore, they were plotted as white. These points can represent alignment angles of 0${}^{\circ }$ or λ/2, for which the intensities of the polarization filters at 45${}^{\circ }$ and 135${}^{\circ }$ are the same. Additionally, the difference in minimum resolution of alignment angle that can be calculated is affected by the depolarization difference between the maximum and minimum wavelengths in the light source.

**Figure 6:**
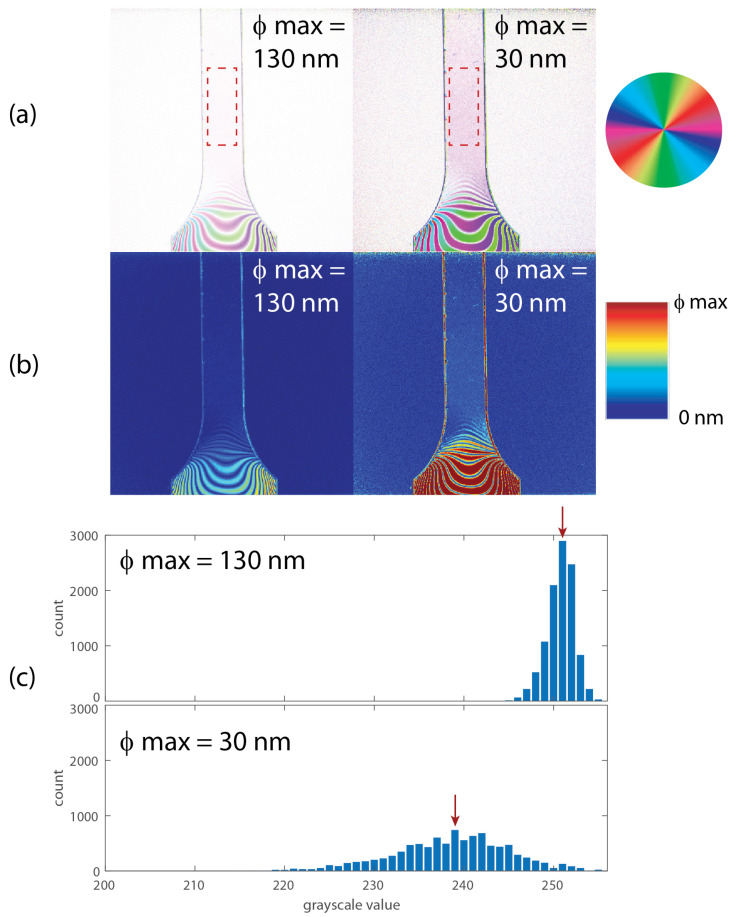
(**a**) Alignment angle and (**b**) retardation images for a polycarbonate specimen using two different maximum retardation angle limits. Region of interest shown as dashed rectangle in (**a**). (**c**) Histograms of pixel distribution in grayscale versions of alignment images for region of interest. Mean pixel value shown with a red arrow. No pixel values were calculated below 200.

To test this assumption, the maximum range of the retardation calculation was varied for a similar specimen. We adjusted the maximum limit of retardation and considered only the pixels for which the calculated retardation was below the set maximum limit. Figure 6 shows the original alignment angle and retardation images collected during the uniform loading period and similar images after the retardation range was reduced to 30 nm (approximately λ/17). Reducing the range increased the number of pixels for which the alignment angle was resolved, as shown in Figure 6a. Figure 6c is a histogram of the pixel distribution (in grayscale values) for the region at the center of the specimen for both maximum retardation ranges. For the original range, the distribution is narrow about the mean value of 251, corresponding to the unresolved alignment angle (plotted as white in the image). Once the maximum retardation was reduced, the mean value shifted to 239 (light pink), closer to the actual alignment angle, with a wider distribution of values due to the measurement noise and calculation uncertainty. Obviously, adjusting this limit removes some of the retardation information, but it does significantly improve the alignment information.

At 9 ms, we could see the sample start to neck at the top. This caused the lower portion of the specimen to move down in the images. The stress field also became a much more complicated distribution of alignment angle and retardation. The expected stress concentration lobes near the notch were visible in the bottom-right hand side of the sample. These lobes were identical to those seen using the rotating polarizer [1]. The plastically deformed material in the necking could not be resolved due to the large changes in material configuration.

In summary, the use of the polarization filter array did permit high-speed polarization imaging of the polycarbonate specimen under dynamic loading conditions. The alignment angle and retardation value images were consistent with the expected behavior of the material and previous measurements using a rotating polarizer. Importantly, there is not a temporal bias in the images, since all four polarization states were collected at the same time. Temporal bias was a significant issue with the previous measurements [1]. Numerical issues were encountered when the alignment angle was near 0${}^{\circ }$; however, these could be resolved by changing the alignment of the camera relative to the specimen prior to testing. While the spatial resolution was reduced by a factor of four compared to the rotating polarizer method (for the same camera pixel resolution), that did not visually reduce the quality of the images for these specimen and was significantly outweighed by the removal of the temporal bias. In the next section, the polarization-filter-array camera was tested on tendon-to-bone insertion specimens, which have much higher levels of intrinsic material scattering.

### 3.2. Tendon-to-Bone Insertion Specimens

**Figure 7:**
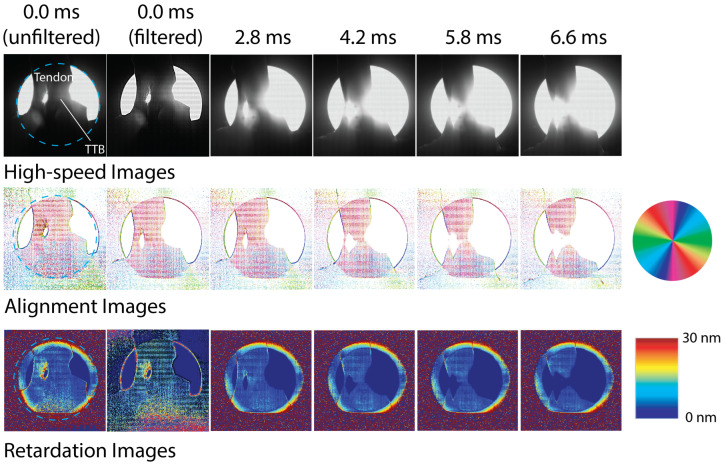
High-speed images recorded for tendon-to-bone insertion specimen 1 using the high-speed polarization-filter-array camera and obtained optical axis alignment angle and retardation images. Field of view is shown as a blue dashed circle in images at 0 ms. Filtered and non-filtered images shown for 0.0 ms. For later time steps, the alignment angle images were filtered; the video and retardation images were not.

**Figure 8:**
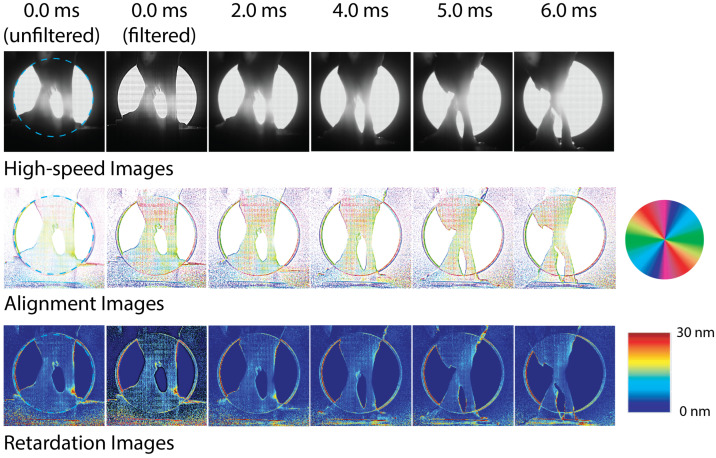
High-speed images recorded for tendon-to-bone insertion specimen 2 using the high-speed polarization-filter-array camera and obtained optical axis alignment angle and retardation images. Field of view is shown as a blue dashed circle in images at 0 ms. Filtered and non-filtered images shown for 0.0 ms. For later time steps, the alignment angle images were filtered; the video and retardation images were not.

Next, we discuss the imaging of the biological tendon-to-bone specimens under the same dynamic loading. As mentioned earlier, the imaging parameters were not the same for these specimens as for the polycarbonate specimens; therefore, we cannot compare the noise levels quantitatively. Instead, we consider the advantages and disadvantages of the polarizing filter array high-speed camera for testing of these materials. As compared to the polycarbonate, biological tissue acts as a birefringent medium due to the presence of aligned collagen fibers. The medium is partially scattering and partially transparent; therefore, the alignment angle corresponds to the alignment angle of the collagen fibers, and the retardation corresponds to the strength of the alignment [13]. Figure 7 and Figure 8 show the intensity images and the generated alignment angle and retardation images for two tendon-to-bone insertion specimens during testing. The images have been cropped to 508 × 508 pixels (video images) and 254 × 254 pixels (alignment angle and retardation maps). Again, several specimens were tested; the examples shown in Figure 7 and Figure 8 are representative specimens. The two specimens were initially slightly different in geometry, due to the slicing process and variations in biological specimens. For both specimens, failure in the insertion region began at around 6.0 ms. For these images, the tendon region is at the top, and the bone is towards the bottom of the image. A detailed description of the rotation of the collagen fibers during tensile impact loading determined from the high-speed polarization imaging data using the rotating polarizer is given in Wu et al. [13]. Here, we will not repeat the detailed analysis but instead focus on comparing the image quality between the current and previous high-speed imaging methods.

The dotted blue line in each of the images in Figure 7 at 0 ms indicates the region on the specimen that was illuminated. The rest of the image is provided and analyzed since the software currently does not have the capability to analyze specific subregions.

The unusual shape of biological specimens means that it was difficult to match the geometry of the camera’s field of view to the region of interest. Therefore, it is common to have areas such as as the bright region in Figure 7 where there is no specimen between the light source and the camera. Since high-intensity lighting was required to pass through the tissue material, there is a strong contrast between the pixels with and without tissue. This was not an issue for the polycarbonate specimen.

Throughout the loading, we again observed a significant number of white pixels (unresolved alignment angles) in the alignment angle images. In this case, the white pixels were due to the high scattering coefficient of the tissue, increasing the noise associated with the calculation of the alignment angle. However, for most of the insertion region, data were collected throughout loading. Additionally, an unsharp mask (radius = 2σ, weight = 0.8) filter was applied to the alignment angle images to better highlight the alignment angles that were resolved. This filtering approach reduced the effects of the overillumination in regions of the field of view. Figure 7 and Figure 8 show alignment angle images at 0.0 ms both with and without the filtering. Applying this filter to the alignment angle images from the polycarbonate specimen did not improve the image quality.

At the start of loading, the collagen fibers are roughly aligned in the vertical direction. Starting at the initial tension on the specimen at 0 ms, we could see the presence of a stress concentration in the left section, which caused rotation of the fibers to approximately −45${}^{\circ }$. This stress concentration eventually led to failure at that location at approximately 4.2 ms. We could also see rotation in a region of the right portion of the tendon-to-bone insertion, starting also at 4.2 ms, which is consistent with more of the load being transferred to the right region once the left region fails. Again, this collagen fiber rotation is an indicator of eventual failure in that region at approximately 5.8 ms. The retardation maps for this specimen are not useful due to the high level of scattering, and therefore, noise in the strength of alignment calculation. An increase in alignment in left region, however, can be seen before failure.

The alignment maps and retardation images for the second specimen, shown in Figure 8, are similar, except that the regions of collagen rotation are more extensive, similarly to previous results obtained using using the rotating polarizer [13]. Again, the collagen fibers showed initial vertical alignment and then rotation prior to failure. The retardation maps show significant changes only at local regions prior to failure.

Finally, some comments should be made on the calibration procedure for the tendon-to-bone specimens. The calibration of the camera system with the tendon-to-bone insertion was difficult due to the high level of scatter in the tissue; historically, high-speed camera imaging tests have been performed on optically clear specimens. The goal of the calibration procedure is to provide a baseline intensity for each pixel in the camera polarization filter array, from which the relative polarization states will be calculated for later images. For the polycarbonate specimens, a single calibration was performed for all the specimens. The challenge with calibrating the camera polarization array for specimens with high levels of random scattering is that there are large local variations between specimens. This effect could be further exacerbated by the specimen’s movement during the dynamic loading.

**Figure 9:**
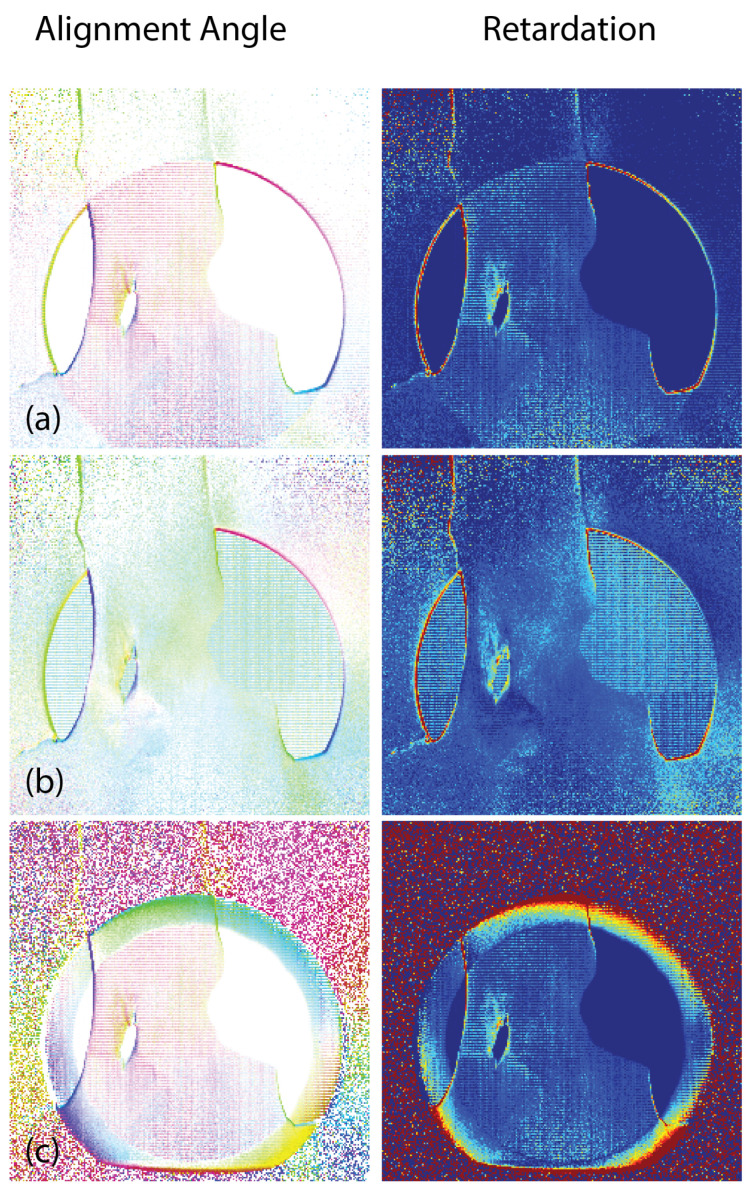
Alignment angle and retardation images for tendon-to-bone specimen 1 at 2.0 ms, calculated using (**a**) original camera settings; (**b**) faster shutter speed; and (**c**) breather sheet with original camera settings. Filtering was not applied to any of the images.

Therefore, we collected calibration-polarization-state images using three different approaches: illuminating the camera imaging area with the same camera settings as during the dynamic tests; illuminating the camera imaging area while reducing the camera shutter speed to reduce the light intensity closer to that transmitted through the tendon-to-bone insertion specimen; and placing a woven fabric breather sheet between the laser illumination and the camera imaging array to better replicate a high-scattering material and using the original camera settings. Figure 9 shows the results of calculating the alignment angle and retardation for specimen 1 at 2.0 ms using each of these calibration data files. The same dynamic test image was used for each case. Despite the fact that the case of Figure 9a is the least close the final illumination, it produced the best alignment and retardation images. This calibration case was used for each of the images in Figure 7 and Figure 8. It is encouraging that the region of the image with the high light intensity (where the light is not passing through the specimen) did not corrupt the rest of the image.

## 4. Conclusions

These experimental results demonstrated the expected advantages of polarization-filter-array methods over the rotating polarizer approach for high-speed imaging of biological tissues under large, dynamic deformations. There was no time lag within a polarization state calculation, as we obtained instantaneous data for each image at that point in time. We were also able to eliminate any motion blur, as there were no moving parts so as long as the shutter speed was short relative to the motion. The method did reduce the overall image resolution by a factor of four. There are software methods to overlap the images using each pixel multiple times and retain the original resolution, only losing information at the borders [17]. However, this is currently not implemented in the commercial software.

The polarization imaging configuration did pose additional challenges due to the need for a calibration of the polarization filter array for a given sample in the same lighting conditions as during the measurement. These experiments showed that the calibration procedure strongly affects the results of the alignment and retardation maps. They also highlighted the potential measurement errors by using a calibration procedure based on a relatively uniform distribution of light intensity. Therefore, the calibration procedure may reduce the sensitivity of the camera to the measurements being obtained in the specimen, and must be thoroughly explored prior to testing of the sample.

Overall, the polarization filter array camera offers some benefits over the rotating polarization camera; however, there are still some other factors, such as alignment, reduction of image size and calibration methods that could use further development.

## Data Availability

The data presented in this study are available on request from the corresponding author.

## Abbreviations

The following abbreviations are used in this manuscript:

LED	Light emitting diode
QWP	Quarter wave plate
